# Watching axons on the move

**DOI:** 10.7554/eLife.106190

**Published:** 2025-02-26

**Authors:** Maria I Lazaro-Pena, Carlos A Diaz-Balzac

**Affiliations:** 1 Division of Endocrinology, Diabetes and Metabolism, University of Rochester Medical Center Rochester United States

**Keywords:** UNC-6/netrin, UNC-40/DCC, axon guidance, haptotaxis, chemotaxis, *C. elegans*

## Abstract

The ligand Netrin mediates axon guidance through a combination of haptotaxis over short distances and chemotaxis over longer distances.

**Related research article** Nichols EL, Lee J, Shen K. 2024. UNC-6/Netrin promotes both adhesion and directed growth within a single axon. *eLife* **13**:RP100424. doi: 10.7554/eLife.100424.

The human brain contains billions of neurons, and the connections between these neurons give rise to networks that are responsible for controlling our behaviors. To understand how a neuron ‘knows’ where to go in order to connect with another neuron, one can think of how we use cars to move around: there are many types of cars (neurons) and many types of roads (brain regions), and car drivers need to abide by a series of rules (cues) to get to their destination (target neuron). The equivalent process in the brain involves a structure called an axon – a long, slender projection from a neuron – being guided to its target (another neuron) by a combination of short-range and long-range cues.

These cues work by means of a ligand that is localized within the extracellular matrix, and a receptor that is located at the tip of the growing axon. Several types of ligand-receptor systems are known, with some ligand-receptor interactions being attractive and others being repulsive (Dickson, 2002). As an axon grows or migrates towards its target, these receptors search for the presence of their respective ligands within the extracellular matrix and either grow towards the ligand or avoid it. However, many aspects of how this process works in vivo are not well understood.

Now, in eLife, Ev Nichols, Joo Lee and Kang Shen of Stanford University report the results of experiments on the worm *C. elegans* that shed new light on the axon guidance ligand called Netrin, which is also known as UNC-6 (Nichols et al., 2024). Previously the Shen laboratory and other research groups have used genetic techniques to investigate the role played by Netrin in axon guidance and other functions in *C. elegans* by studying worms that contained mutant versions of the genes for Netrin and/or its receptor, which is called DCC or UNC-40 (Chan et al., 1996; Colón-Ramos et al., 2007; Hedgecock et al., 1990; Ziel and Sherwood, 2010). Nichols et al. built on these previous experiments by using time-lapse imaging to visualize and quantify in vivo how the axon responded to each genetic intervention as it migrated to its target. The fact that *C. elegans* is translucent and contains just 302 neurons (Brenner, 1974) makes it well suited to such an imaging approach.

Nichols et al. first examined what happens when the axon of the PDE neuron migrates towards its target, which is the ventral nerve cord (Hedgecock et al., 1990; Norris et al., 2014). Surprisingly, they observed that the axon paused for a short time at an intermediary region, before continuing on to the ventral nerve cord and forming a number of branches with it. However, only one of these branches was later stabilized, while the others were discarded (Figure 1).

**Figure 1.**
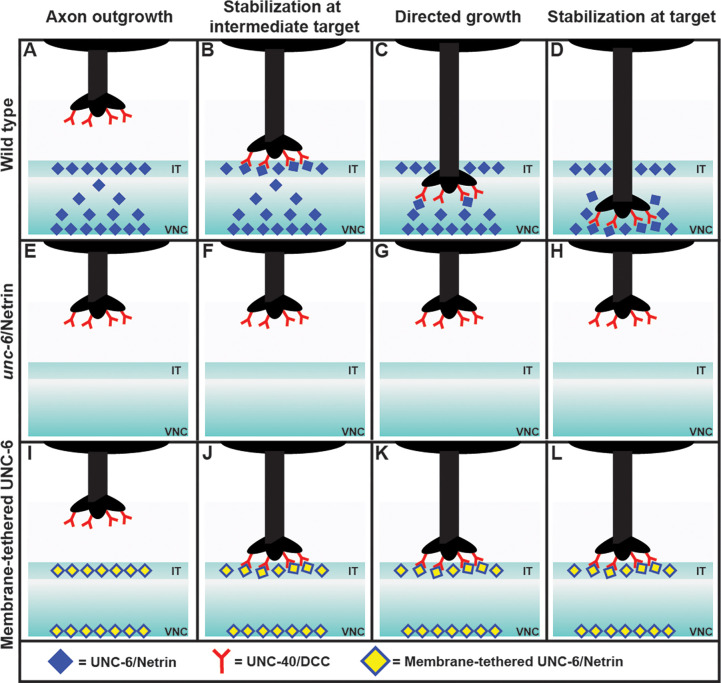
Exploring the axon guidance process in *C. elegans*. In wild-type *C. elegans* (top row), the PDE axon (black) migrates to the ventral nerve cord (VNC) in four steps: axon outgrowth (**A**); stabilization at the intermediate target (IT), with the DCC receptors (red) binding to the Netrin ligands (blue; **B**); directed growth (**C**); and stabilization at target (**D**). The axon forms a number of branches with the VNC, but only one of these becomes stabilized, and the rest are discarded (not shown). In mutant worms lacking Netrin (middle row), the axon does not reach the intermediate target or the ventral nerve cord (**E–H**). Adding a membrane-tethered version of Netrin at the intermediate target and the ventral nerve cord in the mutant worms (bottom row) restores the stabilization at the intermediate target (**J**), but not the subsequent steps (**K, L**). This is consistent with Netrin working as a haptotactic signal to guide the axon to the intermediate target, and as a chemotactic signal to guide the axon from the intermediate target to the ventral nerve cord. When the Netrin is tethered to a membrane it cannot move to establish a concentration gradient, and thus cannot act as a chemotactic signal.

Next they analyzed each step of axon guidance process – axon outgrowth, stabilization at the intermediate target, directed growth, and stabilization at the target – in various mutant worms. As expected, mutants that lacked Netrin exhibited abnormalities at every step (Figure 1). To identify whether Netrin was working as a chemotactic signal (i.e., if the axon was moving in response to changes in the concentration of the Netrin), or as a haptotactic signal (i.e., if the axon was physically adhering to the Netrin), they expressed Netrin tethered to a membrane in the intermediate target region of the mutants.

The researchers found that the first two steps of the process – axon outgrowth and stabilization at the intermediate target – were restored, but subsequent steps were not. This observation showed that Netrin works as a haptotactic signal to stabilize the axon at the intermediate target, and also as a chemotactic signal to allow the axon to grow from the intermediate target to the ventral nerve cord. This conclusion was further supported by the observation of a gradient in the concentration of Netrin in wild type worms, with the ligand being enriched at the intermediate target and also at the ventral nerve cord. A chemical gradient is a classic sign of chemotaxis.

The findings and approach of Nichols et al. set the stage for researchers to investigate what happens during specific steps of axon guidance, and to explore how specific ligand/receptor complexes function within the overall process. Their findings suggest that a given ligand-receptor pair can have more than one mode of action when guiding axons, such as chemotaxis and haptotaxis in the case of Netrin. However, it remains unclear how the axon transitions between these modes of action, and it also remains to be seen if other ligand-receptor interactions have a role in modulating the function of Netrin during the axon guidance process.
